# Cytotoxic Effect of Thymoquinone-Loaded Nanostructured Lipid Carrier (TQ-NLC) on Liver Cancer Cell Integrated with Hepatitis B Genome, Hep3B

**DOI:** 10.1155/2018/1549805

**Published:** 2018-08-16

**Authors:** Aminah Suhaila Haron, Sharifah Sakinah Syed Alwi, Latifah Saiful Yazan, Rohaina Abd Razak, Yong Sze Ong, Fatin Hannani Zakarial Ansar, Henna Roshini Alexander

**Affiliations:** ^1^Department of Biomedical Science, Faculty of Medicine and Health Sciences, Universiti Putra Malaysia (UPM), 43400 Serdang, Selangor, Malaysia; ^2^Laboratory of Molecular Biomedicine, Institute of Bioscience, Universiti Putra Malaysia (UPM), 43400 Serdang, Selangor, Malaysia

## Abstract

Thymoquinone (TQ), a bioactive compound found in* Nigella sativa*, cannot be orally consumed due to its lipophilicity. In order to overcome this low bioavailability, TQ is loaded into a colloidal drug carrier known as a nanostructured lipid carrier (NLC). This study aims to determine the antiproliferative effects of TQ and TQ-NLC on liver cancer cells integrated with the hepatitis B genome, Hep3B. The Hep3B was treated with TQ or TQ-NLC for 24, 48, and 72 hours via MTT assay. The results confirm that TQ or TQ-NLC inhibited the growth of Hep3B at IC50 <16.7 *μ*M for 72 hours. TQ was also found to induce cell cycle arrest at the G1 checkpoint while TQ-NLC induced non-phase-specific cell cycle arrest. Further analysis using Annexin V staining confirmed the apoptotic induction of TQ or TQ-NLC via activation of caspases-3/7. In ROS management, TQ acted as a prooxidant (increased the level of ROS), while TQ-NLC acted as an antioxidant (reduced the level of ROS). Molecular analysis demonstrated that the GSH system and the Nrf2/Keap1 signaling pathway in Hep3B influenced the differential responses of the cells towards TQ or TQ-NLC. Hence, this study demonstrated that TQ and TQ-NLC have* in vitro* anticancer effects on the Hep3B.

## 1. Introduction

Cancer is categorized as a fatal disease caused by the uncontrolled growth of abnormal cells [1]. Hepatocellular carcinoma (HCC) is the sixth most common cancer in humans and the third leading cause of cancer-related death [2]. HCC is a major primary liver cancer subtype that contributes between 70 and 85% to the total incidence of liver cancers worldwide [3]. The incidence of HCC has risen to about 75,000 cases annually worldwide [4]. The causes of HCC include cirrhosis, aflatoxin, heavy alcoholism, nonalcoholic fatty liver disease, Hepatitis B virus (HBV), and Hepatitis C virus (HCV) infection. Among the 250,000 new cases of HCC every year, 80% are caused by HBV and HCV [5]. The Hep3B resembles liver parenchymal disease throughout the growth cycle of the culture, which comprises distinctive rearrangements of chromosome 1 [6]. The Hep3B synthesizes two major polypeptides of the hepatitis B virus surface antigen (HBsAg). Epidemiologically, HBV has been recognized as a contributing agent for the majority of HCC occurrence. The Hep3B will form tumors when injected into nude mice. Hence, it provides an outstanding model for the evaluation of human plasma protein synthesis and the hepatitis B virus-host cell relationship. The Hepatitis B virus X gene is known to be crucial for viral replication and hepatocarcinogenesis [7, 8].

Many chronic diseases including cancer are pathological problems resulting from oxidative stress. Excessive accumulation of reactive oxygen species (ROS) leads to oxidative damage of cellular macromolecules that in turn leads to neoplastic alteration of cells [9]. However, cytoprotective proteins known as phase-2 detoxification enzymes provide the first line of defense against oxidative stress-induced cellular damage [9, 10]. In resting cells, NF-E2 related factor 2 (Nrf2) is sequestered in the cytoplasm by its inhibitory protein Kelch-like ECH-associated protein-1 (Keap1), which causes degradation of Nrf2. Nrf2 dissociates from Keap1 due to oxidative stress and translocates to the nucleus that interacts with small Maf proteins and binds to the ARE sequences in the promoter region [11–13]. Cytoprotective proteins are induced to oxidative and/or electrophilic stress as a safeguard against excessive ROS-induced cellular damage. Thus, Nrf2 inducers function as prooxidants that generate ROS [14]. With respect to cancer, glutathione metabolism can play both protective and pathogenic roles. This is important for the detoxification of carcinogens, which can affect cell survival. Higher levels of glutathione in tumor cells enable the cell to defend against bone marrow, breast, colon, larynx, and lung cancers [15]. Direct stimulation of Nrf2 and restoration of GSH levels are related to age-related hepatic oxidative stress [16]. Treatment of HCC is still a major challenge because standard chemotherapies using doxorubicin, cisplatin, tamoxifen, leuprorelin, and flutamide are typically not effective [17]. Thus, alternative therapies are now drawing the attention of researchers worldwide.

Natural products are rich sources of cancer chemotherapy drugs, which have clinical potential in the prevention and treatment of cancer [18].* Nigella sativa*, an annual flowering plant native to Mediterranean countries, Pakistan, and India, is also commonly known as black cumin [19]. In the Arabian Peninsula, its seed oil had been used traditionally as herbal medicine for the treatment of lung diseases, arthritis, and hypercholesterolemia [20]. Thymoquinone (2-Isopropyl-5-methylbenzo-1,4-quinone; TQ), an active compound of the black seed, is extensively consumed as a condiment in Ayurvedic medicine. A previous study reported that TQ also exhibits anti-inflammatory and antioxidative effects. It also proved that TQ exerts its antineoplastic effects through different modes of action including inhibition of cell proliferation, induction of apoptosis, cell cycle arrest, generation of reactive oxygen species (ROS), and inhibition of metastasis and angiogenesis [21]. In human breast carcinoma, TQ also shows antiproliferative and proapoptotic effects through its induction on p38 and ROS signaling [22]. However, the clinical use of TQ is limited by its poor aqueous solubility [21], hence leading to poor oral bioavailability [23]. Moreover, the intraperitoneal administration route of TQ is restricted, as this method induces much discomfort, is costly, and has sterility issues.

Drug carrier systems offer great promise in improving the therapeutic effectiveness and safety profile of cancer chemotherapy drugs. The nanostructured lipid carrier (NLC) is one of the colloidal drug carrier systems that offer many advantages including the capability to increase bioavailability of poorly soluble drugs, providing the protection for sensitive active compounds/drugs, and facilitating the controlled release of drugs [24]. NLC was developed as an alternative drug carrier system to circumvent the drawbacks of solid lipid nanoparticles (SLN). NLC, which is composed of a mixture of solid lipid and liquid lipid, has shown increased drug loading compared to SLN [25]. The drug loading capacity of NLC can also be improved by changing its lipid matrix composition. It is also expected that drug expulsion during storage will be reduced due to the imperfect crystal lattice of NLC [26, 27].

As previously described by Ng et al. [28], thymoquinone-loaded nanostructured lipid carrier (TQ-NLC) (PATENT NO: PI201200181) was successfully synthesized using a hot high-pressure homogenization method, yielding excellent physicochemical properties. TQ-NLC has a particle diameter of less than 50 nm, and high encapsulation efficiency with good stability of up to two years [29]. With a particle diameter of less than 50 nm, TQ-NLC offers a large surface area for reaction with its target components, and also minimizes the probability of being phagocytosed by the macrophage in a mononuclear phagocytic system [30, 31]. TQ-NLC has also been proven to exhibit cytotoxicity towards breast cancer cell lines (human estrogen receptor negative breast adenocarcinoma, MDA-MB-231, and human estrogen receptor positive breast adenocarcinoma, MCF-7) and cervical cancer cell lines (human cervical adenocarcinoma, HeLa, and human squamous carcinoma, SiHa) [29]. Moreover, TQ-NLC was found relatively noncytotoxic towards normal cells (3T3-L1 and Vero) [29]. Based on an* in vivo* toxicity study, TQ-NLC showed less toxicity compared to free TQ because the lipid carrier that encapsulates TQ minimizes the toxic effect of the compound [31].

Overall, the above evidences prove that TQ-NLC holds great promise as a potential anticancer agent against several types of cancers. However, the role of TQ-NLC in HCC integrated with the hepatitis B genome has yet to be investigated. This present study investigates the cytotoxicity of TQ and TQ-NLC on Hep3B and the underlying mechanisms involved in the mode of cell death and the level of total GSH, as well as Nrf2/Keap1 and caspase protein expression in both cell lines.

## 2. Materials and Methods

### 2.1. Chemical and Reagents

Dulbecco's Modified Eagle's Medium (DMEM), trypsin-EDTA, antibiotics (penicillin and streptomycin), 3-(4, 5-dimethylthiazol-2-yl)-2, 5-di-phenyltetrazolium bromide (MTT) powder, and trypan blue dye solution were purchased from Nacalai Tesque (Kyoto, Japan). Dimethyl sulfoxide (DMSO) was purchased from Fisher Sc. (UK) and the phosphate buffer saline tablet was purchased from Oxoid (England). Fetal Bovine Serum (FBS) was purchased from iDNA (South America Origin). Propidium iodide was purchased from Sigma (St. Louis, USA). Other kits used were the Glutathione Assay Kit (Cayman, USA) and Annexin V/FITC Kit (BD Bioscience, USA). Primary rabbit antibodies anti-Nrf2 (C-20: sc-720), anti-Keap1 (H-190: sc-33569), caspase-3 (H-277: sc-7148), caspase-7 p20 (H-65: sc-33773), and anti-beta-actin (sc-47778) were purchased from Santa Cruz Biotechnology (CA, USA). Horseradish peroxidized conjugated anti-rabbit (ab6721) was purchased from ABCAM (Cambridge, MA, USA). TQ and TQ-NLC were provided by Assoc. Prof. Dr. Latifah Saiful Yazan, Laboratory of Molecular Biomedicine, Institute of Bioscience (IBS), Universiti Putra Malaysia.

### 2.2. Cell Lines

Human liver cancer cells with detected hepatitis B genome (Hep3B) and a normal skin fibroblast cell line (3T3) were purchased from the American Type Culture Collection (ATCC), USA. The cells were cultured in a complete DMEM medium and incubated at 37°C in an atmosphere containing 5% CO_2_.

### 2.3. Growth Inhibition Assay

Cells at 70%–80% confluency were harvested with trypsin-EDTA. Briefly, 1.0 × 10^5^ cells were seeded in a 96-well plate in 0.05 mL complete DMEM. After overnight incubation to allow cell attachment, TQ or TQ-NLC at various concentrations (0.78 *μ*M to 50 *μ*M) was added to the culture medium. A control without treatment was also included. The assay was later terminated at 24, 48, and 72 hours and the ‘relative cell growth' was measured using the MTT assay. After incubation, a MTT solution (5 mg/mL) was added to each well. Absorbance at 570 nm and the reference wavelength of 630 nm were measured using a microplate reader (Opsys MR, USA) [32].

### 2.4. Detection of Mode of Cell Death

Mode of cell death induced by TQ or TQ-NLC was detected using the FITC-Annexin V Apoptosis Detection Kits II (BD Biosciences) following the manufacturer's instruction. Briefly, 1.0 × 10^5^ cells were seeded with 3 mL media in a 6-well plate. After overnight incubation, the cells were treated with TQ or TQ-NLC (12.5 *μ*M) and incubated again for 24, 48, and 72 hours. The cells were harvested and 100 *μ*L of the cells were transferred into 5 mL FACS tubes. The cells were washed twice with cold PBS and centrifuged at 486 ×*g* for 5 minutes. A Master Mix containing 300 *μ*L of binding buffer, 2.5 *μ*L of 50 *μ*g/mL PI, and 1.25 *μ*L of Annexin V-FITC for each sample was prepared in the dark and 300 *μ*L of the mixture was then added to each sample. The cells were incubated for 15 minutes at room temperature in the dark prior to flow cytometry analysis using the FL1 channel on a BD Biosciences FACSCalibur.

### 2.5. Cell Cycle Analysis

In this analysis, 1.0 × 10^5^ cells were seeded with 3 mL media in a 6-well plate followed by overnight incubation. Next, cells were treated with 12.5 *μ*M TQ or TQ-NLC and incubated again for 24, 48, and 72 hours. Cells were harvested and washed, and 100 *μ*L of the cells were transferred into 5 mL FACS tubes. The cell pellets were later stained using propidium iodide (PI). Analysis was done using flow cytometry on a BD FACSCalibur.

### 2.6. Glutathione Measurement

Cells were plated at a density of 1.0 × 10^5^ cells with 3 mL complete growth media into a 6-well plate. The following day, the cells were treated with 12.5 *μ*M TQ or TQ-NLC or left untreated. The level of GSH was measured using kits from Cayman Chemical Company. Briefly, 50 *μ*L of the cells was added to each well of a 96-well plate. As a standard, 50 *μ*L of GSSG standard was used and added to each designated well. An Assay Cocktail was prepared by mixing the GSH MES buffer, GSH Cofactor mixture (NADP+ and glucose-6-phosphate), GSH enzyme mixture (glutathione reductase and glucose-6-phosphate dehydrogenase in 0.2 mL buffer), and GSH 5,5′ Dithiobis (2-nitrobenzoic acid). 150 *μ*L of the freshly prepared Assay Cocktail was added to each well. The absorbance was measured at 405 nm at five-minute intervals for 30 minutes.

### 2.7. Western Blot Analysis

Briefly, 8.0 × 10^5^ cells were seeded with 8 mL media in a T75 flask followed by overnight incubation. After appropriate treatment, the cells were trypsinized followed by whole cell lysate extraction. The cell lysates were centrifuged and the supernatants were collected. A calculated volume of lysate (20 *μ*g) was mixed with a Laemmli sample buffer, whereby the mixture was resolved by 12% SDS/PAGE gel and then electroblotted onto a PVDF membrane. The membrane was probed with a primary antibody (1:1000) for overnight incubation at 4°C and then washed and incubated with HRP-conjugated secondary antibody (1:10000) for 2 hours at room temperature. The membrane was examined for its chemiluminescence using ECL (GE Healthcare, Little Chalfont, Buckinghamshire, UK). A densitometry analysis of the scanned blots was performed using ImageJ software and the results were expressed as a percentage of control after normalization to *β*-actin.

### 2.8. Data Analysis

All data was analyzed using the software package Prism GraphPad Programme (GraphPad Software). Error bars represent ± the standard error of the mean (SEM) for the data set. Comparisons within groups of data were done using one-way analysis of variance (ANOVA) followed by Dunnet using Statistical Package for Social Science (SPSS) version 21.0. A probability of p < 0.05 was considered statistically significant.

## 3. Results

### 3.1. Cytotoxicity of TQ and TQ-NLC on Hep3B

Table 1 shows the antiproliferative effects of TQ and TQ-NLC on the growth of Hep3B and normal 3T3 using the MTT assay. Both compounds decreased the viabilities of Hep3B in a dose- and time-dependent manner compared to normal 3T3. The IC_50_ values obtained for Hep3B treated with TQ for 24, 48, and 72 hours were approximately 2-fold more toxic compared to 3T3 (16.7, 13.1, and 11.1 *μ*M), while the IC_50_ values obtained for the Hep3B treated with TQ-NLC for 24, 48, and 72 hours were 13.5, 11.4, and 9.20 *μ*M, respectively. The IC_50_ values obtained for the 3T3 treated with TQ and TQ-NLC were > 30 *μ*M. Meanwhile, cisplatin, which represents the positive control, was observed to have a very minimum inhibitory effect on all cell lines, with IC_50_ > 50 *μ*M. A similar toxicity pattern was observed in cells treated with NLC.

### 3.2. TQ and TQ-NLC Induce Apoptosis in Hep3B

To elucidate whether the loss of cell viability induced by 12.5 *μ*M of TQ or TQ-NLC was associated with apoptosis, Annexin V staining was performed. This assay determines the phosphatidylserine turnover from the inner to the outer lipid layer of the plasma membrane. Results show that the percentage of early and late apoptotic cells (Table 2) increased in a time-dependent manner in cells treated with TQ or TQ-NLC. The proportion of Hep3B treated with TQ in early and late apoptosis was 13.4%. Meanwhile, after 72 hours, the proportion of Hep3B treated with TQ-NLC in early and late apoptosis was higher (17%) than those of the control (8%).

### 3.3. TQ and TQ-NLC Induce Cell Cycle Distribution in Hep3B

**Figure 1:**
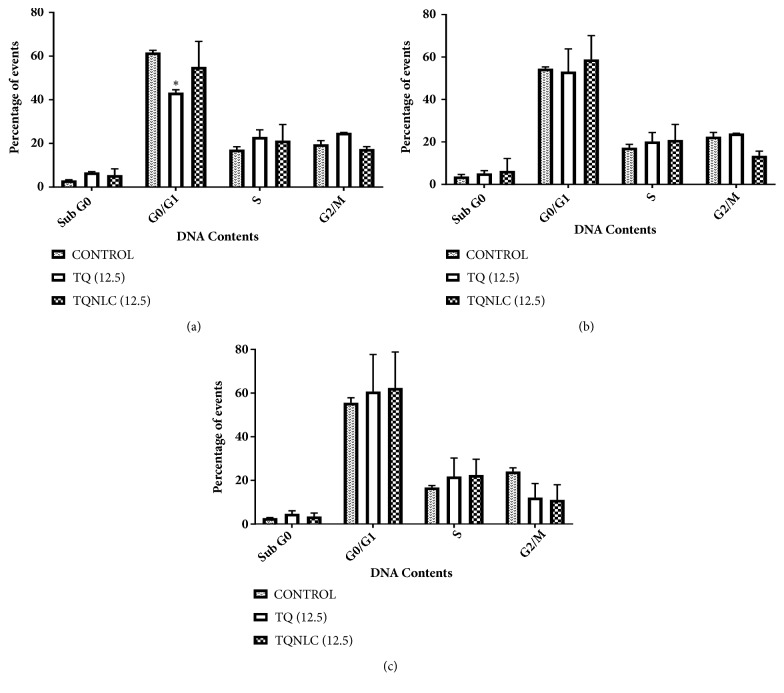
**Cell cycle distribution of Hep3B treated with TQ or TQ-NLC.** (a) 24 hours; (b) 48 hours; and (c) 72 hours. Distribution of cell cycle was assessed by flow cytometry and the data shown are the mean ±SEM of triplicate samples. Statistically significant differences are indicated with (*∗*p<0.05).

To examine the mechanism responsible for TQ and TQ-NLC mediated cell death, cell cycle distribution was evaluated using flow cytometric analysis. The cell cycle arrest of Hep3B by TQ or TQ-NLC was found to be time-dependent (Figure 1). After 24 hours of exposure to 12.5 *μ*M, there was a small increase in the Sub-G0 proportion from 2.5% to 6.5% when treated with TQ and 2.5% to 7.5% when treated with TQ-NLC compared to the control. Based on the histogram analysis, a significant arrest was observed at the G1 phase in the Hep3B treated with TQ with a* p* value < 0.05 compared to the control.

There was a small skew in the data seen from the S phase (26.1%) to G2/M phase (11.9%) in Hep3B treated with TQ-NLC at 48 hours. Although a small increase was observed in Sub-G0, there was no significant phase arrest in the TQ or TQ-NLC compounds. Meanwhile, at 72 hours, cell population in the S phase increased accompanied with a decrease in G2/M population. However, none of these changes were significant compared to the control.

### 3.4. Effect of TQ and TQ-NLC on Caspase-3 and Caspase-7

**Figure 2:**
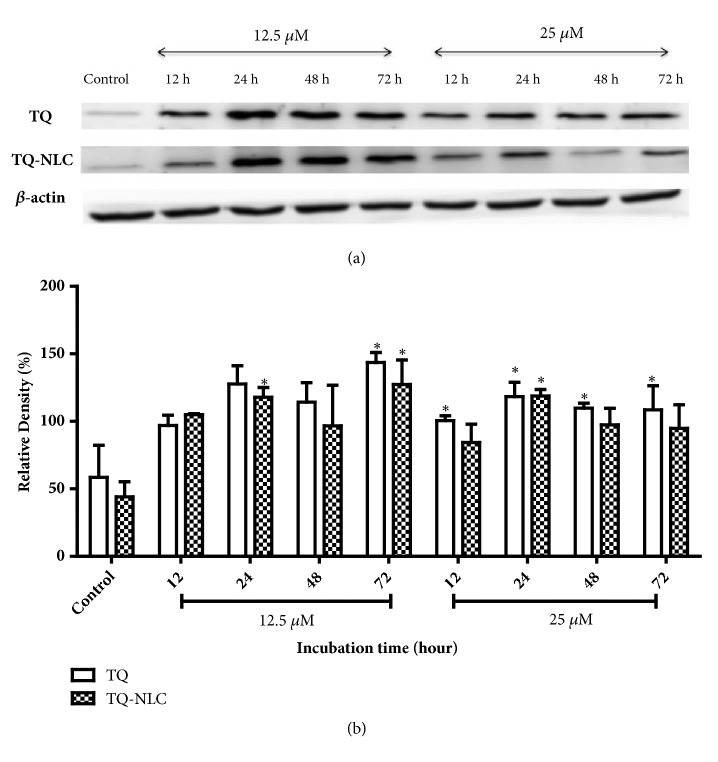
**Caspase-3 expression in Hep3B upon treatment with TQ or TQ-NLC.** (a) Western blot analysis of caspase-3 protein expression after treatment with indicated concentrations of TQ or TQ-NLC for 12, 24, 48, and 72 hours. Whole cell protein lysates were analyzed via Western blotting using antibodies against caspase-3. (b) Protein level was quantified using the densitometry analysis of Image J and expressed as relative density percentage. Data are presented as mean ± SEM and represent three independent experiments. Statistically significant differences are indicated as *∗*p<0.05.** Note: ***∗*compared with the control.

**Figure 3:**
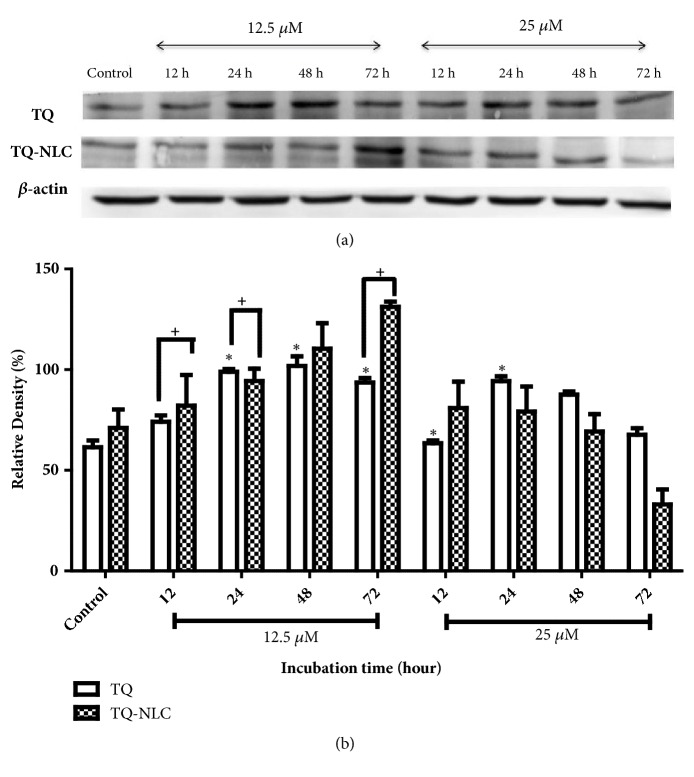
**Caspase-7 expression in Hep3B upon treatment with TQ or TQ-NLC.** (a) Western blot analysis of caspase-7 protein expression after treatment with indicated concentrations of TQ or TQ-NLC for 12, 24, 48, and 72 hours. Whole cell protein lysates were analyzed via Western blotting using antibodies against caspase-7. (b) Protein level was quantified using the densitometry analysis of Image J and expressed as relative density percentage. Data are presented as mean ± SEM and represent three independent experiments. Statistically significant differences are indicated as *∗*p<0.05.** Note: ***∗*compared with control; +compared between TQ and TQ-NLC.

Apoptosis is a complex signaling pathway that mobilizes a number of molecules in response to treatment. To further define the apoptotic mechanism in treated cells, activation of caspase-3 and caspase-7 was investigated in Hep3B treated with TQ and TQ-NLC (Figures 2 and 3, respectively). For protein analysis, a concentration of 12.5 *μ*M for TQ or TQ-NLC was selected to observe the effects of both compounds on protein expression level while 25 *μ*M TQ or TQ-NLC was selected to assess the toxicity of both compounds at higher concentration. Based on the histogram analysis, both TQ and TQ-NLC were observed to significantly activate caspase-3 as early as 24 hours at concentrations of 12.5 *μ*M and 25 *μ*M when compared with the control. However, no significant difference in caspase-3 activation was observed between the compounds and doses given.

Meanwhile, activation of caspase-7 was gradually increased and peaked at 24 up to 72 hours upon treatment with 12.5 *μ*M of TQ. However, at a rather higher concentration (25 *μ*M of TQ), caspase-7 cleavage peaked as early as 12 hours compared to the control and remained at a higher concentration until 48 hours before gradually declining 72 hours after treatment. A similar activation pattern was also observed in Hep3B treated with 12.5 *μ*M TQ-NLC. In contrast, active caspase-7 gradually declined in a time-dependent manner upon treatment with a higher concentration of TQ-NLC (25 *μ*M). A significant difference in response between both compounds was observed at a dose concentration of 12.5 *μ*M for 12, 24, and 72 hours of treatment. This suggests that TQ and TQ-NLC are able to trigger the activation of the intrinsic caspase pathway in Hep3B, i.e., liver cancer cells integrated with a hepatitis B genome model.

### 3.5. Effects of TQ and TQ-NLC on Glutathione

**Figure 4:**
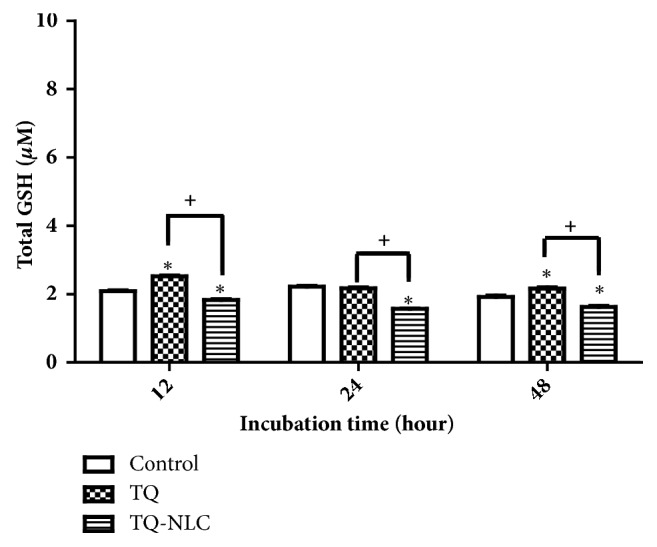
**GSH levels in Hep3B upon treatment with TQ or TQ-NLC**. Data are the mean ± SEM derived from three independent experiments. Statistically significant differences are indicated with (*∗*p<0.05).** Note**: *∗*compared with control; +compared between TQ and TQ-NLC.

The differences in the responses of Hep3B towards TQ or TQ-NLC may be influenced by the encapsulation of TQ in TQ-NLC. Therefore, the level of total GSH in Hep3B was analyzed (Figure 4). Timeframes of 12, 24, and 48 hours were chosen due to the fast turnover of GSH upon activation. Exposure of Hep3B towards TQ significantly increased GSH level at 12 hours after treatment when compared to the control. Although there was a decrease in GSH level at 24 hours, the level increased again after 48 hours of TQ treatment. Interestingly, TQ-NLC exhibited a contradictory effect on GSH level in the Hep3B with a significant reduction observed from 12 to 48 hours after treatment. Moreover, differential responses between these compounds were significant. This suggests that TQ exhibits prooxidant properties while TQ-NLC has a great effect on reducing oxidative stress in Hep3B.

### 3.6. Effect of TQ and TQ-NLC on Nrf2/Keap1

**Figure 5:**
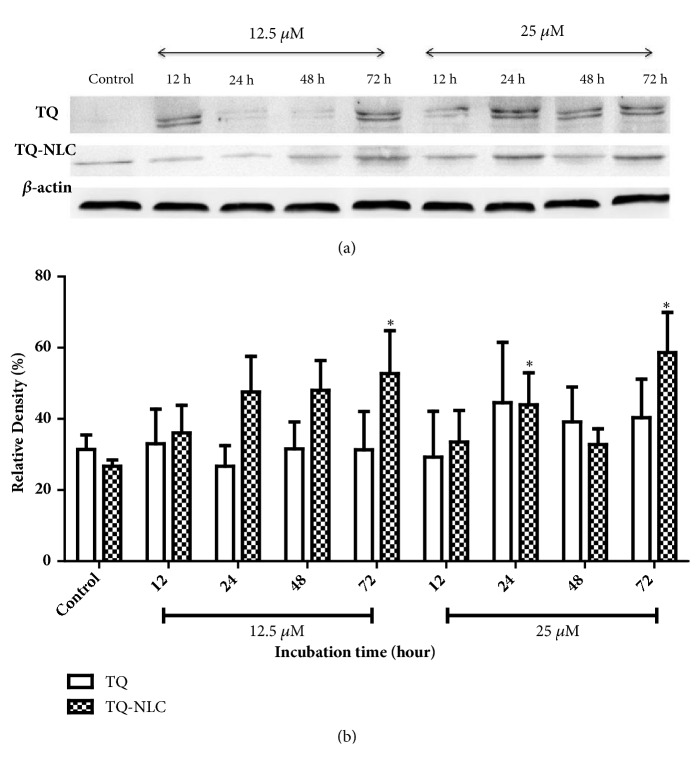
**Nrf2 expression in Hep3B upon treatment with TQ or TQ-NLC.** (a) Western blot analysis of the Nrf2 protein expression after treatment with indicated concentrations of TQ or TQ-NLC for 12, 24, 48, and 72 hours. Whole cell protein lysates were analyzed via Western blotting using antibodies against Nrf2. (b) Protein levels were quantified using the densitometry analysis of Image J and expressed as relative density percentage. Data are presented as mean ± SEM and represent three independent experiments. Statistically significant differences are indicated as (*∗*p<0.05).** Note: ***∗*compared with control.

**Figure 6:**
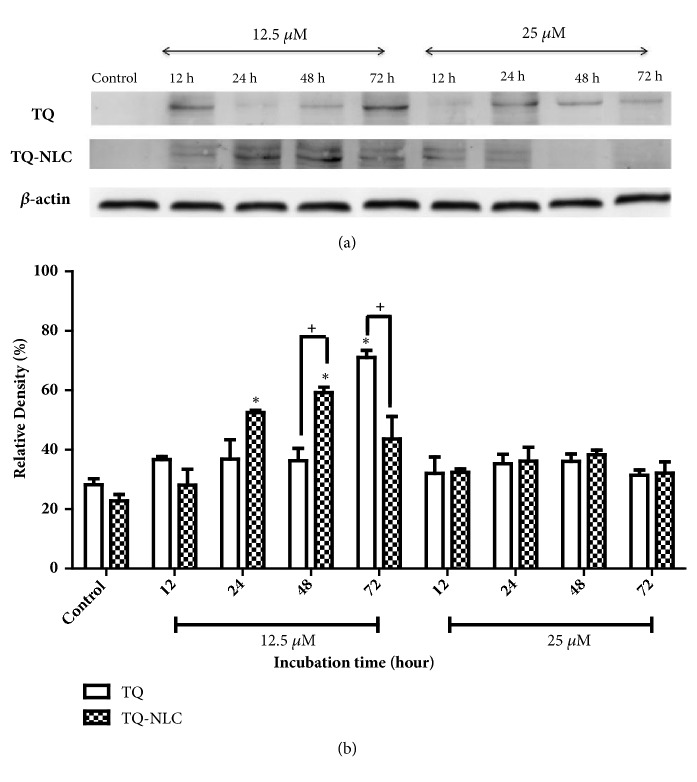
**Keap1 expression in Hep3B upon treatment with TQ or TQ-NLC.** (a) Western blot analysis of Keap1 protein expression after treatment with indicated concentrations of TQ or TQ-NLC for 12, 24, 48, and 72 hours. Whole cell protein lysates were analyzed via Western blotting using antibodies against Keap1. (b) Protein levels were quantified using the densitometry analysis of Image J and expressed as a percentage of relative density. Data are presented as mean ± SEM and represent three independent experiments. Statistically significant differences are indicated as (*∗*p<0.05).** Note: ***∗*compared with control; +compared between TQ and TQ-NLC.

Despite the increased level of GSH observed in Hep3B treated with TQ, the contradictory effect of TQ-NLC suggests that the encapsulation of TQ may play a role in the difference in oxidative stress status triggered by both compounds. Therefore, to confirm these findings, Nrf2 expression as a surrogate marker of oxidative stress was analyzed. Although there was a gradual increase in Nrf2 from 24 to 72 hours in Hep3B treated with TQ (12.5 *μ*M), no significant increase was observed when compared to the control. Similarly, TQ-NLC also increased the level of Nrf2 with 72 hours marking a significant increase compared to the control. At higher concentrations of TQ-NLC, a significant increase in Nrf2 was observed as early as 24 hours before it reduced and increased again at 72 hours. Although there was a fluctuation in Nrf2 expression at 48 hours, this phenomenon might be due to the response towards the instability of ROS production (Figure 5). Notably, the Nrf2 expression frequently looks like a double band in low percentage SDS-PAGE gels (<7.5%). However, the cause of this phenomenon is yet to be verified scientifically [33]. Meanwhile, Keap1 expression increased significantly at 24 and 72 hours in Hep3B treated with TQ. A gradual increase in Keap1 in Hep3B treated with TQ-NLC (12.5 *μ*M) was observed as early as 24 hours and peaked at 48 hours. Keap1 expression remains static at higher concentrations (25 *μ*M) of TQ or TQ-NLC throughout the experiment (Figure 6). Therefore, it is suggested that the sensitivity of Hep3B towards TQ and TQ-NLC is dose- and time-dependent and may be influenced by the level of ROS in the treated cell.

## 4. Discussion

Thymoquinone is an active compound derived from* Nigella sativa*, which is known for its anticancer properties in diverse* in vitro* and* in vivo* human cancer cell models [34, 35]. The encapsulation of TQ in NLC has increased drug efficiency and controlled the drug release. Although TQ-NLC has been reported to be more cytotoxic towards breast cancer MDA-MB-231 cells and cervical cancer SiHa cells compared to TQ [28], studies have yet to determine the toxicity of TQ-NLC towards the liver cancer cell line model, i.e., Hep3B. This model is widely used as cellular reference to develop new drugs and to gain insights into drug metabolism. The cell line differs in number of chromosomes. Moreover, Hep3B is a hepatitis B virus positive and is tumorigenic [6, 36]. They often exhibit different responses of cytotoxicity, gene expression induction, and cell cycle response as well as biochemical effects when given the same pharmacological treatment under the same experimental conditions [37]. Therefore, it is a great advantage to highlight the cytotoxicity effects of TQ, and its improved bioavailability product, TQ-NLC, on Hep3B.

Although numerous studies have reported the antiproliferative effect of TQ on several cancer cells such as human breast and adenocarcinoma cells [38], myeloblastic leukemia cells, HL-60 [39], prostate and pancreatic cancer cells [40, 41], and laryngeal neoplastic cells-Hep-2 [42], no study has yet reported the effect of either TQ or TQ-NLC on liver cancer integrated with the hepatitis B genome, Hep3B. As shown in Table 1, TQ-NLC was found to be more potent towards Hep3B compared to TQ. Although the exact mechanism that causes this differential response towards TQ and TQ-NLC is still unknown, the encapsulated form of TQ may be one of the factors that contribute to this differential effect, as it also improved bioavailability and cytotoxicity. Thus, a key observation for this work is to focus on the differential effect of TQ and TQ-NLC on Hep3B. Our data shows that TQ and TQ-NLC are less toxic towards normal 3T3, with IC_50_ > 30 *μ*M. Similar cytotoxic selective effects of both compounds were also observed on normal cells 3T3-L1 and Vero at 72 hours posttreatment, with IC_50_ values of > 50 *μ*M and 32 *μ*M, respectively [43, 44].

Further molecular analysis of our work showed that Hep3B were relatively sensitive towards TQ and TQ-NLC (78%–85% of healthy cells; 13%–18% of apoptotic cells). Similar evidence of reduced cell viability was also demonstrated in breast, colon, bone, and leukemia cells. The classical hallmarks of apoptosis such as chromatin condensation, translocation of phosphatidyl serine across plasma membrane, and DNA fragmentation were also observed in TQ-treated cells [45]. TQ has also been reported to activate the mitochondrial/intrinsic pathway via release of cytochrome C from mitochondria into the cytosol that later binds to Apaf-1 (apoptosis protease activation factor-1). This leads to the activation of initiator caspase-9, which was observed in human myeloblastic leukemia HL-60 cells [39] and PC cells upon exposure to TQ [40].

The cell cycle analysis results demonstrated a significant arrest at the G0/G1 phase after 24 hours of treatment with TQ. Although the exact underlying mechanism for this effect is unknown, TQ was reported to induce G1 arrest in mouse papilloma carcinoma cells by inducing the expression of p16 and decreasing the cyclin D1 expression level. Moreover, TQ has been documented as capable of inhibiting cell proliferation and malignant transformation from tumor cells through G1/S phase arrest thereby inhibiting the progression of HCC [46]. Most cell cycle regulation proteins such as cyclin D1, E, and CDK4 complexes are essential and are mainly involved in the G1/S phase transition during cell cycle in normal cells. Overexpression of these cyclins can alter cell cycle progression, which leads to malignancy [47]. Therefore, inhibition of cyclin-CDKs complexes may block carcinogenesis development via the arrest of G1/S phase dysregulated cell cycle progression. On the contrary, no significant cell cycle arrest was demonstrated in Hep3B treated with TQ-NLC although an increase in cell cycle arrest was noticed at the Sub-G0 phase at 24 hours and S phase at 48 hours. TQ-NLC was also found to induce non-phase-specific cell cycle arrest in MDA-MB-213 cells at different exposure time. Although there was no explicit explanation, it is postulated that the cyclin-dependent kinases (CDKs) are the main orchestrators of this event [28].

We also examined the caspase-3/7 activity in Hep3B to determine the mechanism of apoptosis of the TQ- and TQ-NLC-treated cells. Proapoptotic proteins, including cytochrome C, were released from the intermembrane space into the cytosol via activation of the BCL-2 family members Bax and Bak [48–50]. Cytochrome C can then bind Apaf-1, forming the apoptosome and activating caspase-9, which directly activates caspase-3 and caspase-7 [51, 52]. Caspase has been shown to activate during apoptosis in many tumor cells. Activation of caspase-3, which is essential for DNA fragmentation, represents a key and irreversible point in the development of apoptosis irrespective of the pathway (mitochondrial or cell death pathway) [53]. TQ has also been reported to activate the mitochondrial/intrinsic pathway via the release of cytochrome C from mitochondria into the cytosol that later binds to Apaf-1 (apoptosis protease activation factor-1). This leads to the activation of initiator caspase-9, which was observed in human myeloblastic leukemia HL-60 cells and PC cells upon exposure to TQ [40]. As reported by Acharya [54], the activation of caspase-3 and subsequent PARP cleavage after TQ treatment indicates that TQ might follow the intrinsic pathway of mitochondrial apoptosis in A549 cells. Meanwhile, caspase-7 is considered to be redundant when used with caspase-3 because these associated cysteine proteases have several common endogenous protein substrates and share an optimal peptide recognition sequence [55]. Therefore, based on the histogram analysis, our indicated experimental doses of 12.5 *μ*M and 25 *μ*M of TQ and TQ-NLC demonstrated significant activation of both caspases-3/7, which led to apoptotic induction via the intrinsic mitochondrial pathway.

Oxidative stress has been confirmed as one of the main contributors to the development of hepatocarcinogenesis and promotion of liver cancer [56]. High levels of oxidative stress later induce the master antioxidant regulator, Nrf2. Nrf2 then regulates a set of encoding proteins involved in the cytoprotein as well as the GSH system [57]. In Hep3B, TQ is suggested to act as a prooxidant before it reduces the level of oxidative stress in the subsequent time exposure. This small increase in ROS triggered by TQ as exogenous stimuli may induce cell death since almost all cancer cells are usually under oxidative stress with a relatively high basal level of ROS [58, 59]. Although TQ is known as an antioxidant at low concentrations, it has also been reported to promote intracellular ROS, promoting DNA damage-related apoptosis through the p73-dependent mitochondrial and cell cycle signaling pathway in Jurkat cells [60, 61]. Increased ROS accumulation was also observed in HCC cells exposed to TQ (6-100 *μ*M) [62]. As reported previously, TQ mediates ROS production by promoting oxidative/electrophilic stress via quinone-quinol redox cycles, which acts as a mechanism to induce apoptosis in various cancer cells [63]. This is due to the presence of quinone derivatives that are capable of inducing cellular ROS generation, which depends on redox potential. Quinone also represents an important class of oxidative and electrophilic molecules [64]. TQ, which is considered as a redox-cycler, can be metabolized into a semiquinone radical by oxidoreductases. Semiquinone acts as a prooxidant that generates ROS such as superoxide anion and general free radical scavenger [65, 66]. Therefore, semiquinone that is generated from metabolism of TQ could lead to ROS production in Hep3B. This explains the increased GSH level in Hep3B treated with TQ, which is essential for reducing the ROS level triggered by TQ. GSH is a tripeptide antioxidant that directly scavenges ROS in the cells with two molecules of reduced GSH generating oxidized glutathione (GSSG) [67]. Hence, it is thought that GSH acts in concert with Nrf2 as antioxidants to reduce oxidative stress level although the increased Nrf2 status upon TQ treatment was insignificant compared to the control. Therefore, from the high GSH and Nrf2 levels in the control cells, we postulate that Hep3B has a high basal ROS level. The subsequent elevated ROS induction by TQ may push the ROS level over the threshold and therefore induce cell death. The fluctuation in GSH level at 48 hours may be associated with the inconsistent ROS reaction with redox-cycler, the TQ compound that influences ROS level. Moreover, the half-life of free radicals is very short, causing limitation in the diffusion distances [68]. This leads to the variable effects of ROS inducers [69].

Nonetheless, TQ-NLC shows a contradictory effect in Hep3B with a significant reduction of GSH level of up to 24 hours after treatment. Nrf2, on the other hand, was upregulated as early as 12 hours after TQ-NLC treatment. Thus, we speculate that the increased Nrf2 expression is due to quinone, which serves as an electrophile, enabling it to act as an indirect antioxidant leading to its gene induction [70]. ROS has been found in various chronic diseases. It may not be the result of disease damage but could be the response of the host to the disease [22, 71]. Therefore, our work suggests that the ROS level in the liver cancer cell models may influence the response in both compounds. Thus, the pathway for which TQ or TQ-NLC mediates its ROS production has to be further explored.

## 5. Conclusion

In conclusion, we suggested that TQ and its improved bioavailability product; TQ-NLC may be an effective antiproliferative agent for treatment of liver cancer. Our data showed that both compounds significantly inhibited the proliferation of Hep3B in a time- and dose-dependent manner which was accompanied by the activation of caspases-3 and -7. However, both compounds responded differently towards induction of cell cycle arrest as well as modulation of Nrf2 and GSH levels that was governed by the ROS production in Hep3B. Therefore, further investigations on the mechanisms underlying the anticancer effects of both compounds (TQ and TQ-NLC)* in vitro* and* in vivo* are needed to map out detailed pathways as well as explain such differences.

## Figures and Tables

**Table 1:** Cytotoxic effects of TQ and TQ-NLC on the Hep3B and 3T3 reflected by the IC_50_ values as determined by the MTT assay.

Cell line	Incubation time (hours)	IC_50_ (*μ*M)				
		TQ	TQ-NLC	DMSO	CISPLATIN	NLC
3T3	24	38.1 ± 0.95	34.4±5.92	NP	NP	NP
	48	32.1 ± 5.20	34.4±5.92	NP	NP	NP
	72	31.5 ± 1.70	34.4±5.92	> 50	> 50	> 50

Hep3B	24	16.7 ± 2.86	13.5±3.58	NP	NP	NP
	48	13.1 ± 0.12	11.4±0.72	NP	NP	NP
	72	11.1 ± 0.41	9.20±2.25	> 50	> 50	> 50

Percentage of viable cell computed in comparison to untreated cells calculated as a percentage. Each value was represented as the mean ±SEM. NP: not performed.

**Table 2:** Percentage of viable, apoptotic, and necrotic Hep3B as determined by flow cytometer.

	Percentage of cell			
	Viable Cell	Early apoptosis	Late apoptosis	Necrosis
UNTREATED	89.1 ± 0.8	4.3 ± 0.7	3.7 ± 0.1	2.9 ± 1.1
NLC	88.9 ± 2.8	3.9 ± 1.1	2.5 ± 1.1	4.7 ± 2.0
TQ (24 hours)	84.5 ± 0.9	6.0 ± 1.4	6.9 ± 0.5	2.6 ± 0.4
TQ (48 hours)	83.4 ± 0.4	7.1 ± 1.3	6.0 ± 0.9	3.4 ± 0.3
TQ (72 hours)	82.0 ± 3.3	7.1 ± 0.9	6.4 ± 0.5	4.5 ± 1.2
TQ-NLC (24 hours)	81.1 ± 1.3	6.7 ± 1.6	6.0 ± 0.7	6.2 ± 2.5
TQ-NLC (48 hours)	79.8 ± 3.2	6.9 ± 1.3	7.7 ± 0.7	5.6 ± 0.9
TQ-NLC (72 hours)	78.2 ± 2.0	9.3 ± 0.5	8.0 ± 1.4	4.7 ± 0.6

Each data point represents the mean of three independent experiments. Each value was represented as a mean ±SEM.

## Data Availability

The data used to support the findings of this study are included within the article.
